# Preparation of Cu-Al/SiO_2_ Porous Material and Its Effect on NO Decomposition in a Cement Kiln

**DOI:** 10.3390/ma13010145

**Published:** 2019-12-30

**Authors:** Yanling Gan, Suping Cui, Xiaoyu Ma, Hongxia Guo, Yali Wang

**Affiliations:** College of Materials Science and Engineering, Beijing University of Technology, Beijing 100124, China; ganyanling1102@126.com (Y.G.); maxiaoyu@bjut.edu.cn (X.M.); hxguo@bjut.edu.cn (H.G.); wangyali1978@bjut.edu.cn (Y.W.)

**Keywords:** Cu-Al/SiO_2_ porous material, template, NO decomposition rate, cement kiln, denitrification mechanism

## Abstract

Nitrogen oxide (NOx) emissions have attracted much attention for increasing concern on the quality of the atmospheric environment. In view of NO decomposition in the cement production process, the preparation of Cu-Al/SiO_2_ porous material and its effect on NO decomposition were studied, and the denitrification mechanism was proposed in this paper. The NO decomposition performance of the Cu-Al/SiO_2_ porous material was tested via the experimental setup and infrared spectrometer and micro gas chromatography (GC). The result shows that the Cu-Al/SiO_2_ porous material with the template of cetyltrimethylammonium bromide (CTAB) had a better NO decomposition rate than materials with other templates when the temperature was above 500 °C, and NO decomposition rate could approach 100% at high temperatures above 750 °C. Structure analysis indicates that the prepared Cu-Al/SiO_2_ material structure was a mesoporous structure. The X-Ray Diffraction (XRD) and Ultraviolet–visible spectrophotometry (UV–Vis) results of the denitrification product show that the Cu-Al/SiO_2_ material mainly decomposed to Cu_2_O and Si_2_O, and the CuO decomposed to Cu_2_O and O_2_ at high temperature. The Cu(I)O was considered as the active phase. The redox process between Cu(II)O and Cu(I)O was thought to be the denitrification mechanism of the Cu-Al/SiO_2_ porous material.

## 1. Introduction

Nitrogen oxides (NOx), as one of the main atmospheric pollutants, are extremely toxic to human health and are also harmful to the environment, which can result in acid rain, photochemical smog, ozone depletion, atmospheric visibility degradation, and so on [1,2,3]. The main source of nitrogen oxides is the burning of chemical raw materials, mainly including nitrogen oxides from industrial production and motor vehicle exhaust fumes [4,5]. The nitrogen oxides from industrial production mainly include coal-fired power plants and industrial furnaces. Nowadays, The NOx emissions from the cement industry become the third-largest one following that from the thermal power plants and motor vehicle exhausts, with the amount of NOx approximately one-tenth of the total emissions [6]. Consequently, reducing NOx emissions from the cement industry has attracted much more public attention in recent years [7,8].

In cement production, there are three kinds of NOx production mechanisms taking place during combustion processes, they are fuel NOx, thermal NOx, and prompt NOx [9]. In addition, NOx is primarily formed at two positions in kiln systems for cement clinker production: In the rotary kiln and the precalciner. NOx is formed both from atmospheric N_2_ and from fuel-bound nitrogen at high temperature in the rotary kiln, whereas, in the precalciner, NOx can be only formed from fuel-bound nitrogen, because of the low operating temperatures [10,11]. At present, denitrification technologies that usually be applied to cement kilns include low NOx combustion technology [12], selective catalytic reduction (SCR) [13,14], and selective non-catalytic reduction (SNCR) [15]. Thermal NOx formation can be decreased by approximately 30% after low NOx combustion technology [16,17], so the efficiency of low NOx combustion technology is too low to meet the emission standard of air pollutants for cement industry. The selective non-catalytic reduction technology (SNCR) is relatively simple and cost-effective technology for removing NOx. SNCR is regarded as the most suitable technology in consideration of the denitrification performance and cost [18]. The SNCR technology for utility boilers has been well studied, and the denitrification efficiency is approximately 40% [19]. Nevertheless, as opposed to SNCR used in power station boiler, NOx reduction efficiencies vary from 15% to 80% between projects, and even within individual projects, for cement kilns [15,18]. The reason is that the SNCR process can be influenced by multiple factors in the complex environment of the cement precalciner. Besides, large amounts of ammonia and urea would be injected into precalciner, ammonia escape results in a secondary pollution. The selective catalytic reduction (SCR), a high-efficiency method, is inclined to be relatively expensive, either to install and run due to expensive consumable [20,21]. Furthermore, the catalysts are easy poisoning and clogging when used in cement kiln, because of high dust concentration and alkali metal content in the exhaust gas from cement kiln [22]. Even integrated usage of above technologies (except for SCR, which is too expensive for cement industry) cannot meet the increasingly stringent emission standard. Therefore, there are no mature technologies to remove NOx in cement industry until now. Given the noted situation, a new NOx emission control technology applied in cement industry was proposed in this study.

In order to effectively reduce NOx emission in the dry process cement kiln with preheater and precalciner, when the rotary kiln exhaust gas containing a large amount of NOx (about 1000 ppm) enters into the precalciner, a kind of porous material can be used as the denitrification catalyst to be injected into the preheater and precalciner without ammonia or other reductant, and this material promotes the decomposition of NOx.

Combined with the cement kiln process, the denitrification porous material would be injected into the cement kiln with raw cement material, so it could not influence the cement performance. Therefore, the component of denitrification material should be compatible with the cement components. The main components of cement are CaO, SiO_2_, Al_2_O_3_, FeO, and so on. Thus, with Al/SiO_2_ as the frame and Cu as the active element, Cu-Al/SiO_2_ porous materials were prepared in this paper. In addition, the effect on the NO decomposition rate was studied in this paper.

## 2. Materials and Methods

### 2.1. Preparation of Porous Materials

The Cu-Al/SiO_2_ porous materials were synthesized by hydrothermal method. In addition, the Cu-Al/SiO_2_ porous materials were prepared with different templates such as cetyltrimethylammonium bromide (CTAB), tetrapropylammonium hydroxide (TPAOH), ammonium hydroxide (NH_3_·H_2_O), and zeolite seed crystal in this paper.

The sodium hydroxide solution was dropped into silica sol under vigorous stirring with a magnetizer (solution A). In addition, the template was mixed with the aluminum sulfate solution (solution B). After stirring for half an hour, solution B was slowly dropwise added into the solution A; finally, the copper sulfate solution was added. The obtained molar composition of gel mixture was SiO_2_: 0.02Al_2_O_3_: 0.05CuO: 0.1CTAB: 0.3NaOH: 45H_2_O. During the process, sulfuric acid solution was used to ensure that the pH of the solution was about 10~12. The solution was stirred at room temperature for 3 h, and poured into a reaction kettle, then the reaction kettle was put in an oven at 160 °C for 24 h. After that, the reaction kettle was cooled in the air, the solid product was separated by centrifugation, and washed with deionized water and ethanol 3 times. The final product was dried at 100 °C for 24 h and then calcined at 550 °C for 5 h.

### 2.2. Characterization

**Figure 1:**
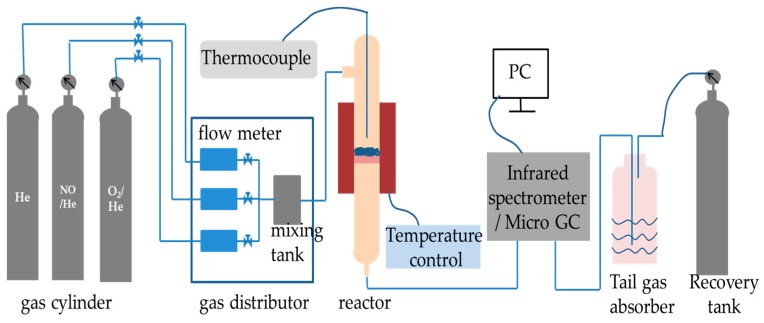
The experimental setup for decomposition of NO.

The NO decomposition reaction was carried out in a laboratory-scale quartz fixed bed reactor heated by an electric control heated oven, as shown in Figure 1. NO decomposition reaction was performed on a fixed-bed quartz tube reactor containing 2 mL porous material. The thermocouple was inserted into the center of the reactor to measure the test temperature. The total flow rate was 300 mL/min. The reactant gas condition was 1000 ppm NO and He balance gas. The materials and reactant gas were heated at a heating rate of 15 °C/min from ambient temperature to 1000 °C. The concentrations of NO, NO_2_, and N_2_O were continually monitored by a TENSOR 27 FT-IR spectrometer (Bruker, Karlsruhe, Germany). In addition, the NO decomposition rate was calculated by Equation (1):

(1) $$\mathrm{NO}\;\mathrm{decomposition}\;\mathrm{rate}\;=\left({\left[\mathrm{NO}\right]}_{\mathrm{in}}-{\left[\mathrm{NO}\right]}_{\mathrm{out}}-{\left[{\mathrm{NO}}_{2}\right]}_{\mathrm{out}}-{\left[N_{2}O\right]}_{\mathrm{out}}\right)/{\left[\mathrm{NO}\right]}_{\mathrm{in}}\times 100\%$$

The specific surface area, N_2_ adsorption desorption curves, and pore size distribution were determined by BET using a TriStarII 3020 gas sorption analyzer (Mike, Georgia, USA). All the samples were fully dried at 90 °C for 1 h and then degassed at 350 °C for 3 h in vacuum prior to the BET surface measurements.

The crystal structures of the prepared porous materials were determined by X-ray diffraction (XRD). XRD patterns were recorded on a Bruker D8 Advance diffractometer (Bruker, Karlsruhe, German) equipped with the Cu-Kα radiation source (λ = 0.15405 nm), operating at 40 kV and 40 mA. In addition, the scanning angle (2θ) was taken over a range of 10°–60°. The diffraction patterns were used for phase identification of crystalline materials or crystalline grains in powder. Crystalline phases were identified by comparison with the reference data cited from the International Center for Diffraction Data (ICDD) files. However, it should be reminded that this technique can only evaluate the crystalline phases and any amorphous phases could not be detected. The TEM was obtained with JEOL-2010 transmission electron microscope (JEOL, Tokyo, Japan). The porous material powders were dispersed in alcohol and kept in an ultrasonic bath for 10 min, and then the well-prepared suspension was deposited on a carbon-covered Cu grid to be tested. UV–Visible diffuse reflectance spectra of the samples were recorded using a JASCO V-650 spectrophotometer (JASCO, Tokyo, Japan).

## 3. Results and Discussion

### 3.1. Denitrification Performance and Structure

**Figure 2:**
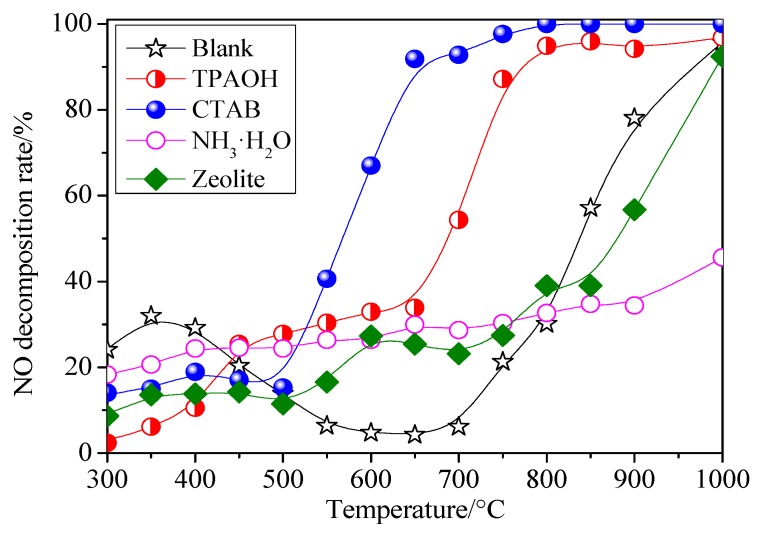
The effect of porous materials with different templates on the NO decomposition rate (Blank, tetrapropylammonium hydroxide (TPAOH), cetyltrimethylammonium bromide (CTAB), ammonium hydroxide (NH_3_·H_2_O), and Zeolite were used, respectively, instead of the Cu-Al/SiO_2_ porous materials with no template added, TPAOH, CTAB, NH_3_·H_2_O, and zeolite seed crystal).

Figure 2 displays the relationship between NO decomposition rate of the porous materials and the temperature ranging from 300 to 1000 °C, with the denitrification data collected in an interval of 50 °C. The porous materials were prepared with different templates (TPAOH, CTAB, NH_3_·H_2_O, zeolite seed crystal and no template added) by hydrothermal method. It can be seen that the NO decomposition rate of Cu-Al/SiO_2_ porous material increased with the increasing of the denitrification reaction temperature. Cu-Al/SiO_2_ porous material with CTAB and TPAOH have better denitrification efficiency than Cu-Al/SiO_2_ porous material with NH_3_·H_2_O and zeolite seed crystal during the temperature range 500–1000 °C. The porous material with CTAB had more than 60% NO decomposition rate at 600–1000 °C, while the NO decomposition rate at 300−500 °C was lower than 20%, and the NO decomposition rate could approach 99% at high temperatures above 750 °C. However, the porous material with TPAOH could reach 60% NO decomposition rate at 750–1000 °C. Compared with the Cu-Al/SiO_2_ porous material with TPAOH, NO decomposition rate of the Cu-Al/SiO_2_ porous material with CTAB was increased by 63.1% and 10.7% at 650 and 750 °C, respectively, indicating that the denitrification efficiency was enhanced with a broader operation temperature window due to the template of CTAB. On the whole, the NO decomposition rate of the Cu-Al/SiO_2_ porous material with the templates of CTAB had the best denitrification efficiency, compared to that of porous materials with other templates.

**Figure 3:**
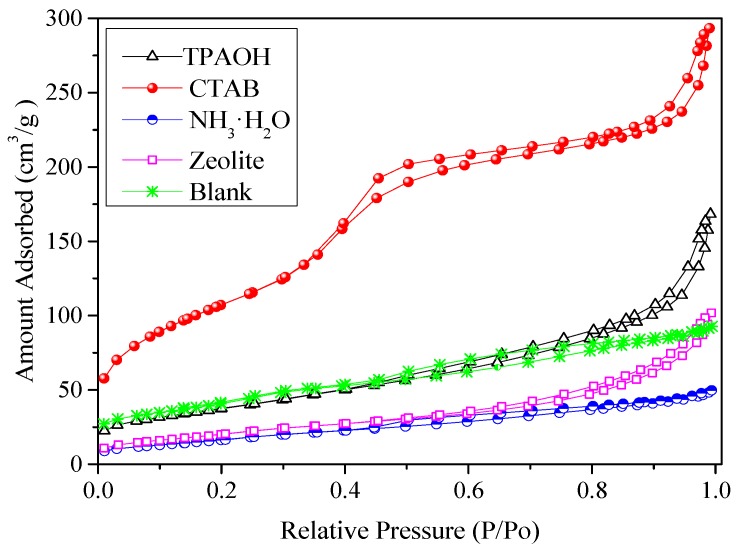
The N_2_ adsorption–desorption isotherm of Cu-Al/SiO_2_ porous materials with different templates (Blank, TPAOH, CTAB, NH_3_·H_2_O, and Zeolite were used, respectively, instead of the Cu-Al/SiO_2_ porous materials with no template added, tetrapropylammonium hydroxide (TPAOH), cetyltrimethylammonium bromide (CTAB), ammonium hydroxide (NH_3_·H_2_O), and zeolite seed crystal).

The Cu-Al/SiO_2_ materials have different porous properties with different templates. Thus, the pore properties of the Cu-Al/SiO_2_ porous materials with varying templates were obtained from N_2_ adsorption–desorption isotherms at −196 °C. The results of BET surface area, pore volume, and pore diameter estimated by the Barrett–Joyner–Halenda (BJH) model are presented for Cu-Al/SiO_2_ porous materials with different templates in Table 1. The results show that the porous materials with organic template reagent had higher specific surface area than those with inorganic template agent. The Cu-Al/SiO_2_ porous material with CTAB reached the largest specific surface area, the biggest cumulative pore volume, and the smallest average pore diameter. The specific surface area, pore volume, and the pore diameter were about 392.27 m^2^/g, 0.4876 cm^3^/g, and 4.3119 nm, respectively. Compared with the Cu-Al/SiO_2_ porous material with CTAB, the specific surface area of Cu-Al/SiO_2_ porous material with TPAOH was decreased to 135.27 m^2^/g, and the pore diameter was increased to 7.55 nm. Figure 3 shows the N_2_ adsorption–desorption isotherms of Cu-Al/SiO_2_ porous materials with no template added, TPAOH, CTAB, NH_3_.H_2_O, and zeolite seed crystal, separately. The template provides key functions in the process of synthesizing the pore structure material, namely in the roles of structure orientation, space filling, and balancing skeleton charge, and so on. From Figure 3, we can see that the N_2_ amount absorption of the Cu-Al/SiO_2_ porous material with CTAB was much larger than those of materials with other templates. The pore structure of Cu-Al/SiO_2_ porous materials was confirmed by N_2_ adsorption–desorption isotherms. The N_2_ adsorption–desorption isotherm of porous material with CTAB shows that the adsorption–desorption hysteresis loop appeared in the middle segment. The Cu-Al/SiO_2_ porous material with CTAB had the typical IV type N_2_-adsorption isotherm curves, and the material had the inflection point and hysteresis loop, implying the material’s pores were well distributed and narrow (also shown in the pore size distribution curve, the distribution of pore diameter was mainly in the range 2~5 nm), which indicate that the material structure was a mesoporous structure. Thus, the pore structure of the Cu-Al/SiO_2_ porous material with CTAB enlarged the contact area with NO gas, which contributed to the denitrification performance.

**Figure 4:**
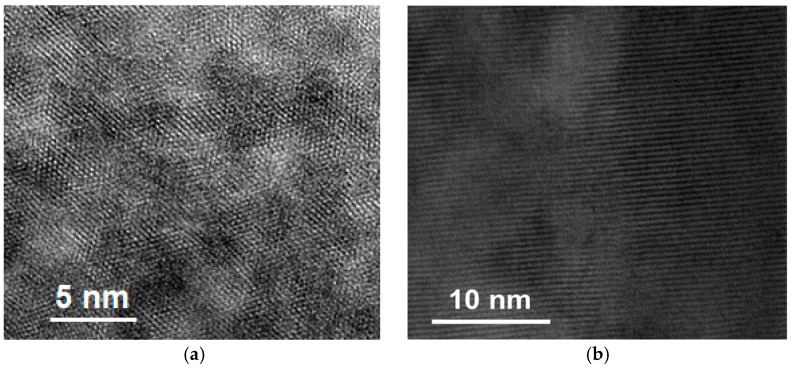
The transmission electron microscope (TEM) micrograph of Cu-Al/SiO_2_ porous material with cetyltrimethylammonium bromide (CTAB). (**a**) parallel to the pore direction; (**b**) perpendicular to the pore direction.

A transmission electron microscope (TEM) was used to study the microstructure of Cu-Al/SiO_2_ porous material with the template of CTAB (as shown in Figure 4). The TEM micrograph in Figure 4 demonstrated the uniform one-dimensional mesoporous channel structure of Cu-Al/SiO_2_ porous material. It was notable that the uniform porosity illustrated by TEM analysis was in line with the quite narrow pore size distribution determined by N_2_ adsorption (see Figure 3). Representative TEM images of the Cu-Al/SiO_2_ porous material with a highly ordered hexagonal structure could be clearly observed in the image taken with the electron beam parallel to the pore direction (Figure 4a). Additionally, the micrograph that was taken with the electron beam perpendicular to the pores showed both the channels and the framework (Figure 4b). The well-ordered mesoporous structure was observed clearly and it could hardly find nanoparticles or clusters of metal (Cu) oxides outside the mesoporous channels, indicating the copper was incorporated into the Si-O and Al-O framework in Cu-Al/SiO_2_ material.

As the component of the Cu-Al/SiO_2_ porous material was similar to the cement composition, it did not negatively influence the performance of cement. In combination with the cement production process, it is suitable to add the Cu-Al/SiO_2_ porous material into the preheater with the cement raw materials. NO would be removed in the preheater and precalciner. The products of the denitrification reactions goes into the rotary kiln with the cement raw material, and, finally, contributes to the cement clinker in the rotary kiln. During the whole process, the Cu-Al/SiO_2_ porous material is not only a denitrification material but also can be a component of the cement clinker.

**Figure 5:**
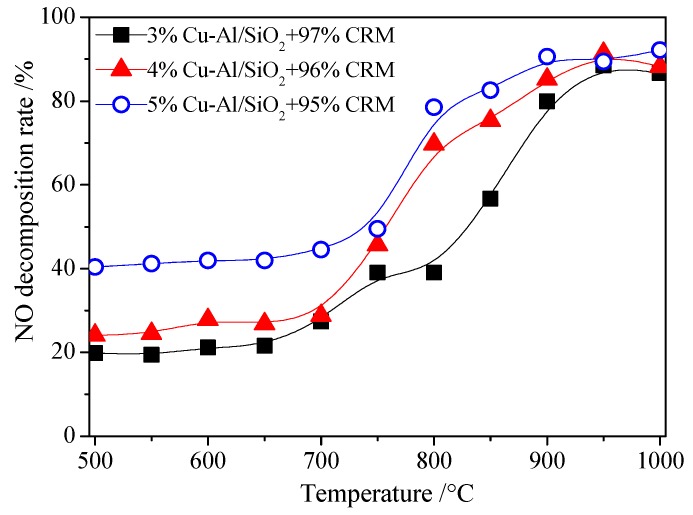
Effect of adding different amounts of Cu-Al/SiO_2_ material in cement raw materials (CRM) on NO decomposition rate.

In order to ensure the denitrification performance of the Cu-Al/SiO_2_ porous material in cement kiln, cement raw materials (CRM) mixed with low content (3%~5%) of Cu-Al/SiO_2_ porous materials with the template of CTAB were tested. It can be seen, from Figure 5, that the NO decomposition rate increased with the increase of the proportion of the Cu-Al/SiO_2_ porous material in the mixture material. In addition, the NO decomposition rate increased with the increase of the temperature from 500 to 1000 °C. When a small amount of Cu-Al/SiO_2_ porous material (5%) was added into cement raw materials, the NO decomposition rate of the mixture material was more than 80% at 800–1000 °C, while the NO decomposition rate was lower than 50% at 500−700 °C. NO decomposition rate increased quickly when the temperature was above 750 °C, and the decomposition rate of all the materials could reach as high as 80% above 900 °C. When a small amount of Cu-Al/SiO_2_ porous material (5%) was added into cement raw materials, the NO decomposition rate could reach over 80% above 800 °C, and the NO decomposition rate could approach 90% at the temperature of 900 °C.

### 3.2. Denitrification Mechanism

**Figure 6:**
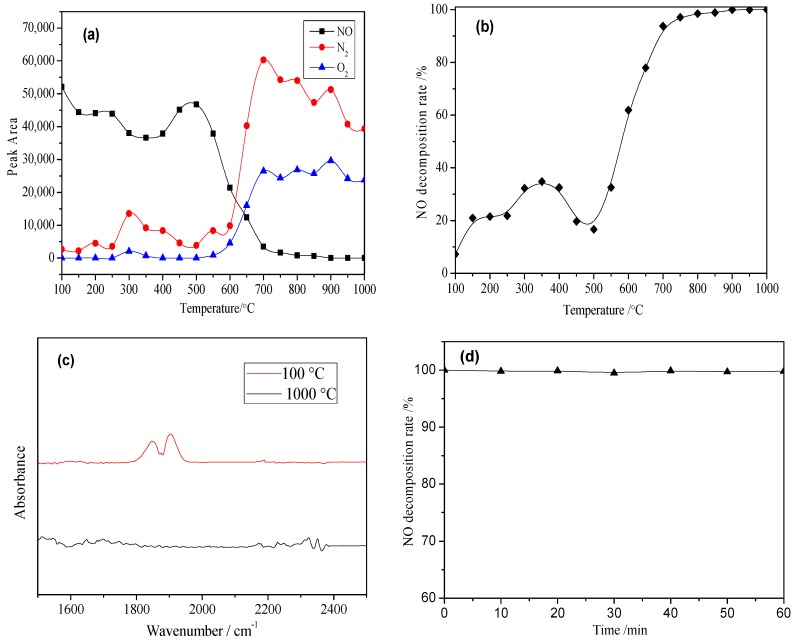
Denitrification results of porous material with CTAB tested by micro gas chromatography (GC) and Fourier Transform Infrared Spectroscopy (FTIR). (**a**) Peak area of different gases after denitrification; (**b**) NO decomposition rate of porous material; (**c**) FTIR spectra of reaction gas after denitrification at different temperatures; (**d**) NO decomposition rate of porous material when the temperature is kept at 1000 °C for 60 min.

According to the results of the denitrification performance of Cu-Al/SiO_2_ porous materials with different templates (TPAOH, CTAB, NH_3_·H_2_O and ZSM seed crystal), the Cu-Al/SiO_2_ porous material with CTAB obtained the best NO decomposition rate above 500 °C. Therefore, in order to further study the denitrification mechanism of the Cu-Al/SiO_2_ porous material, under the gas condition of 1000 ppm NO and balance gas He, experiments were carried out with fixed bed reactor system, and the reaction gas after denitrification was analyzed with GC (Agilent 490 Micro GC, California, USA). The NO decomposition rate of the Cu-Al/SiO_2_ porous material with the template of CTAB was calculated by Equation (1). In addition, the gas flow rate was 100 mL/min, and the reaction temperature was increased from 100 to 1000 °C at 15 °C/min. The denitrification result of the Cu-Al/SiO_2_ porous material with CTAB is shown in Figure 6.

The amount of different gas is proportional to the peak area, thus Figure 6a illustrates that peak area of different gases changed along with the temperature. As can be seen in Figure 6a, the amount of NO decreased sharply and, correspondingly, the amount of N_2_ and O_2_ increased at high temperature (above 500 °C). In addition, there was basically no NO_2_ and N_2_O produced in the whole process. The result tested by micro GC is coincident with that of detected by FTIR (as shown in Figure 6c). There was NO absorption peak (1803~1962 cm^−1^) in reaction gas after denitrification at 100 °C. In addition, the NO absorption peak disappeared and basically no NO_2_ absorption peak (1550~1650 cm^−1^) and N_2_O absorption peak (2160~2262 cm^−1^) appeared in reaction gas after denitrification at 1000 °C. According to the result of Figure 6a, NO decomposition rate could be calculated by Equation (1). Therefore, NO decomposition rate of Cu-Al/SiO_2_ porous material with the template of CTAB was shown in Figure 6b. NO decomposition rate reached to nearly 40% at low temperature of 350 °C, and increased quickly above 500 °C. In addition, the NO decomposition rate of Cu-Al/SiO_2_ porous material with CTAB could approach 100% at high temperatures above 750 °C. After the denitrification test from 100 to 1000 °C, NO decomposition rate of Cu-Al/SiO_2_ porous material was tested when the temperature was kept at 1000 °C for 60 min, as shown in Figure 6d. The gaseous products of the reaction were analyzed by micro GC. The GC result shows that there was no nitrogen oxides left in the gaseous products, thus NO decomposition rate was still nearly 100% in the 60 min.

**Figure 7:**
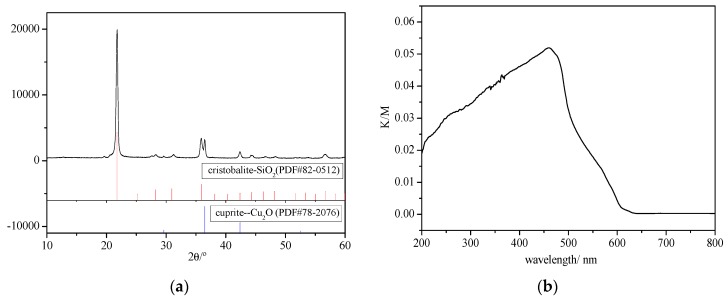
The analysis of the Cu-Al/SiO_2_ porous material with CTAB after denitrification reaction. (**a**) The X-ray diffractometer (XRD) analysis; (**b**) The Ultraviolet–visible spectroscopy (UV–Vis) analysis.

The phase analysis of denitrification product was analyzed by X-ray diffractometer and Ultraviolet–visible spectroscopy (UV–Vis) (shown in Figure 7). It can be seen from Figure 7a, XRD phase analysis indicates that the denitrification products mainly were cristobalite and cuprite. The XRD pattern of cuprite revealed main diffraction peaks at 2θ values of 29.7°, 36.6°, and 42.5°. These values correlated with the cubic crystalline phase of Cu_2_O according to PDF reference number 78-2076. The XRD pattern of cristobalite showed main diffraction peaks at 2θ values of 21.7°, 28.2°, 30.9°, 35.9°, and 46.3°. These values correlated with the tetragonal crystalline phase of Si_2_O according to PDF reference number 82-0512.

The result of the UV–Vis absorbance analysis (Figure 7b) reveals that the maximum absorbance of Cu_2_O in the visible light range (400–800 nm) occurred at around 470 nm, which is consistent with nanometer Cu_2_O [23,24,25]. The results of XRD and UV–Vis indicate that during the increasing of the temperature, the porous structure of the Cu-Al/SiO_2_ porous material collapsed, and Cu-Al/SiO_2_ porous material decomposed to Cu_2_O, Si_2_O, and very little aluminum oxide. CuO in the porous material decomposed to Cu_2_O and O_2_ at high temperature.

The results of XRD and UV–Vis analysis show that the CuO in Cu-Al/SiO_2_ porous material decomposed to Cu_2_O and O_2_, and then the material reached a high NO decomposition rate at high temperature. Therefore, the Cu(I)O was considered as the active phase, and the denitrification mechanism of the Cu-Al/SiO_2_ porous material was proposed, being that the NO decomposition is a redox process between Cu(II)O and Cu(I)O [26]. Several excellent reviews [27,28,29] have been published for establishing the reaction mechanism of NO decomposition over Cu-zeolites. The denitrification mechanism of the Cu-Al/SiO_2_ porous material is thought to be similar to that of the Cu-zeolites in this paper. As for the reaction mechanism of the NO decomposition, various experimental and theoretical models have been suggested, and considerable evidence has been provided to indicate that Cu(I) species participate in the NO decomposition reaction [30,31,32,33,34]. There are some discrepancies among the concepts of the form of the intermediate active sites. However, in our opinion, there is no doubt that the NO decomposition is a redox process.

**Figure 8:**
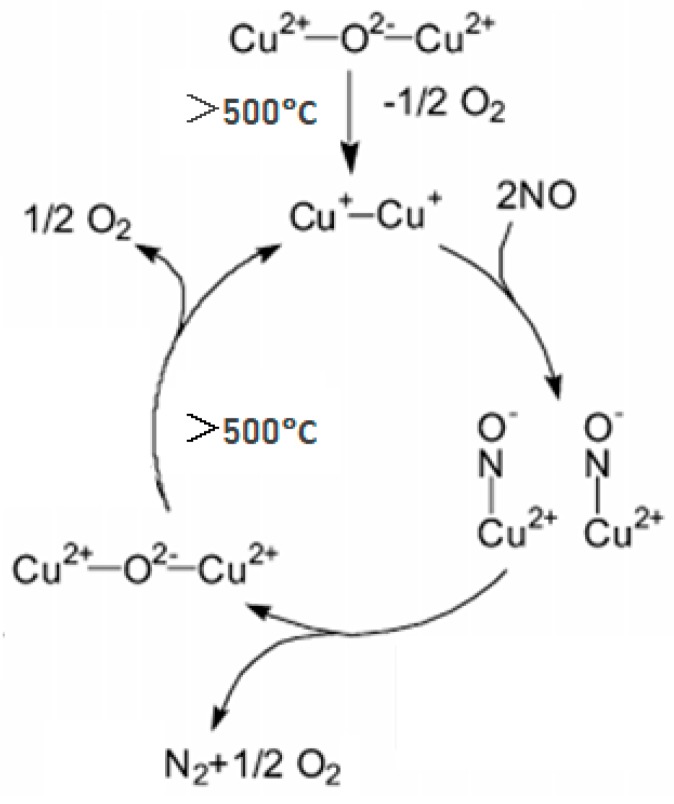
Proposed denitrification mechanism of the Cu-Al/SiO_2_ porous material at high temperature.

Thus, Figure 8 described the proposed denitrification mechanism of the Cu-Al/SiO_2_ porous material at high temperature [31]. As we can see in Figure 8, during the process of heating, a pair of Cu(II) ions would lose an oxygen ion above 500 °C, a pair of Cu(I) ions were generated, and a Cu(I) active center was formed. Then, two NO molecules were adsorbed on the pair of Cu(I) ions, and an electron of Cu(I) ion was easily lost and went into the NO antibonding orbital, which formed an intermediate of adsorbed anion pairs (Cu^2+^-NO^−^). NO molecules took one electron from the Cu(I) ions, then bond length of N−O became longer, and the bond of N–O became easier to break, and finally the anion decomposed to N_2_ and O_2_ molecules. Cu(II) ion was formed due to losing an electron of the Cu(I) ion. Then Cu(I) ions active site was re-generated from the Cu(II) ion easily at temperatures above 500 °C. This denitrification mechanism is consistent with experimental results, thus the denitrification mechanism of Cu-Al/SiO_2_ was thought to be that the NO decomposition is a redox process between Cu(II)O and Cu(I)O.

## 4. Conclusions

The Cu-Al/SiO_2_ porous material with the template of cetyltrimethylammonium bromide (CTAB) had a better NO decomposition rate than materials with other templates when the temperature was above 500 °C, and the NO decomposition rate could approach 100% at high temperatures above 750 °C. The structure analysis indicates that the prepared Cu-Al/SiO_2_ material structure was a mesoporous structure, and average pore diameter was 4.3119 nm. When adding a small amount of Cu-Al/SiO_2_ porous material (5%) into cement raw materials, the NO decomposition rate still could reach over 80% above 800 °C. In addition, the NO decomposition rate increased with the incremental addition of proportions of Cu-Al/SiO_2_ porous material. The XRD and UV–Vis results of the denitrification product show that the Cu-Al/SiO_2_ porous material mainly decomposed to Cu_2_O and Si_2_O. The CuO in the Cu-Al/SiO_2_ porous material decomposed to Cu_2_O and O_2_ at temperatures above 500 °C. The Cu(I)O was considered as the active phase. The redox process between Cu(II)O and Cu(I)O was thought to be the denitrification mechanism of the Cu-Al/SiO_2_ porous material.

## Figures and Tables

**Table 1:** The results of specific surface areas, pore volumes, and pore diameters of porous materials with different templates (Blank, TPAOH, CTAB, NH_3_·H_2_O, and Zeolite were used, respectively, instead of the Cu-Al/SiO_2_ porous materials with no template added, tetrapropylammonium hydroxide (TPAOH), cetyltrimethylammonium bromide (CTAB), ammonium hydroxide (NH_3_·H_2_O), and zeolite seed crystal).

Template	Specific Surface Area (m^2^/g)	Cumulative Pore Volume (cm^3^/g)	Average Pore Diameter (nm)
CTAB	392.2759	0.4876	4.3119
TPAOH	135.2742	0.2702	7.5585
Blank	124.693	0.1396	4.674
Zeolite	77.5880	0.1619	7.6493
NH_3_·H_2_O	64.5010	0.0813	4.5998

## References

[1] Lv G., Lu J.D., Cai L.Q., Xie X.H., Liu Z.X. (2011). Experimental study on the dynamic process of NO reduction in a precalciner. Ind. Eng. Chem. Res..

[2] Chen L.Q., Niu X.Y., Li Z.B., Dong Y.L., Dong Wang D., Yuan F.L., Zhu Y.J. (2016). The effects of BaO on the catalytic activity of La_1.6_Ba_0.4_NiO_4_ in direct decomposition of NO. J. Mol. Catal. A Chem..

[3] Zhu J., Thomas A. (2009). Perovskite-type mixed oxides as catalytic material for NO removal. Appl. Catal. B Environ..

[4] Fang D., Li D., He F., Xie J.L., Xiong C.C., Chen Y.L. (2019). Experimental and DFT study of the adsorption and activation of NH_3_ and NO on Mn-based spinels supported on TiO_2_ catalysts for SCR of NOx. Comput. Mater. Sci..

[5] Koebel M., Elsener M., Kleeman M. (2000). Urea-SCR: A promising technique to reduce NOx emissions from automotive diesel engines. Catal. Today.

[6] Zhang L.J. (2015). Study on the Preparation and Performation of MnO_x_/TiO_2_ DeNOx Catalytic Materials at Low Temperature Used in Cement Kiln. Ph.D. Thesis.

[7] Kawakami M., Furumura T., Tokushige H. (2007). Proceedings of International RILEM Symposium on Photocatalysis.

[8] Battye R., Walsh S., Lee-Greco J. (2000). NO_x_ Control Technologies for the Cement Industry.

[9] Mahmoudi S., Baeyens J., Seville J. (2010). NOx formation and selective non-catalytic reduction (SNCR) in a fluidized bed combustor of biomass. Biomass Bioenerg..

[10] Fan W.Y., Zhu T.L., Sun Y.F., Lv D. (2014). Effects of gas compositions on NOx reduction by selective non-catalytic reduction with ammonia in a simulated cement precalciner atmosphere. Chemosphere.

[11] Huang L., Lu J.D., Hu Z.J., Wang S.J. (2006). Numerical simulation and optimization of NO emissions in a precalciner. Energy Fuel..

[12] Hu W.F., Xie J.M. (2009). Feasibility study on the low NOx combustion transformation of a 600 MW unit boiler. Electr. Power Constr..

[13] Zhang L.J., Cui S.P., Guo H.X., Ma X.Y., Luo X.G. (2015). The poisoning effect of potassium ions doped on MnO_x_/TiO_2_ catalysts for low-temperature selective catalytic reduction. Appl. Surf. Sci..

[14] Yim S.D., Kim S.J., Baik J.H., Nam I.S., Mok Y.S., Lee J.H., Cho B.K. (2004). Decomposition of Urea into NH_3_ for the SCR Process. Ind. Eng. Chem. Res..

[15] Fu S.L., Song Q., Tang J.S., Yao Q. (2014). Effect of CaO on the selective non-catalytic reduction deNOx process: Experimental and kinetic study. Chem. Eng. J..

[16] Li C., Cui S.P., Gong X.Z., Meng X.C., Wang H.T. (2013). LCA Method of MSC and Low-NOx Burner Technology in cement manufacturing. Mater. Sci. Forum..

[17] Bebar L., Kermes V., Stehlik P., Canek J., Oral J. (2002). Low NOx burners-prediction of emissions concentration based on design, measurements and modelling. Waste Manag..

[18] Shammakh M.S. (2011). A decision support tool formulti-pollutants reduction incement industry using analytic hierarchy process (AHP). Can. J. Chem. Eng..

[19] Fu S.L., Song Q., Yao Q. (2015). Study on the catalysis of CaCO_3_ in the SNCR deNOx process for cement kilns. Chem. Eng. J..

[20] Strege J.R., Zygarlicke C.J., Folkedahl B.C., Mccollor D.P. (2008). SCR deactivation in a full-scale cofired utility boiler. Fuel.

[21] Benson A.S., Laumb D.J., Crocker R.C., Pavlish H.J. (2005). SCR catalyst performance in flue gases derived from subbituminous and lignite coals. Fuel Process. Technol..

[22] Yang B., Zheng D.H., Shen Y.S., Qiu Y.S., Li B., Zeng Y.W., Shen S.B., Zhu S.M. (2015). Influencing factors on low-temperature deNOx performance of Mn–La–Ce–Ni–Ox/PPS catalytic filters applied for cement kiln. J. Ind. Eng. Chem..

[23] William W.A., Sudheesh K.S., Poomani P.G. (2018). Graft Gum Ghatti Caped Cu2O Nanocomposite for Photocatalytic Degradation of Naphthol Blue Black Dye. J. Inorg. Organomet Polym. Mater..

[24] Zhang X.X. (2010). Controlled Synthesis and Photocatalytic Activity of Cuprous Oxides. Master’s Thesis.

[25] Bernard C.H., Fan W.Y. (2006). Shape Evolution of Cu_2_O Nanostructures via Kinetic and Thermodynamic Controlled Growth. J. Phys. Chem. B.

[26] Kuroda Y., Iwamoto M. (2004). Characterization of cuprous ion in high silica zeolites and reaction mechanisms of catalytic NO decomposition and specific N_2_ adsorption. Top. Catal..

[27] Centi G., Perathoner S., Shioya Y., Anpo M. (1992). Role of the nature of copper sites in the activity of copper-based catalysts for no conversion. Res. Chem. Intermed..

[28] Centi G., Nigro C., Perathoner S. (1994). Specific activity of copper species in decomposition of NO on Cu-ZSM-5. React. Kinet. Catal. Lett..

[29] Iwamoto M., Yahiro H., Tanda K., Mizuno N., Mine Y., Kagawa S. (1991). Removal of nitrogen monoxide through a novel catalytic process: Decomposition on excessively copper-ion-exchanged ZSM-5 zeolites. J Phys. Chem..

[30] Iwamoto M., Yahiro H. (1994). Novel catalytic decomposition and reduction of NO. Catal. Today.

[31] Yahiro H., Iwamoto M. (2001). Copper ion-exchanged zeolite catalysts in deNO*x* reaction. Appl. Catal. A Gen..

[32] Ma T., Wang R. (2008). Catalytic Decomposition of NOx. Prog. Chem..

[33] Li H.L., Xiao P., Wang T., Zhu J.J., Li J.L. (2014). Recent progress on catalysts used for NO decomposition. Sci. Sin. Chim..

[34] Garin F. (2001). Mechanism of NO*x* decomposition. Appl. Catal. A Gen..

